# Development of a Traceability System Based on a SNP Array for Large-Scale Production of High-Value White Spruce (*Picea glauca*)

**DOI:** 10.3389/fpls.2017.01264

**Published:** 2017-07-25

**Authors:** Julie Godbout, Laurence Tremblay, Caroline Levasseur, Patricia Lavigne, André Rainville, John Mackay, Jean Bousquet, Nathalie Isabel

**Affiliations:** ^1^Natural Resources Canada, Canadian Forest Service, Laurentian Forestry Centre Québec, QC, Canada; ^2^Direction Générale de la Production de Semences et des Plants Forestiers, Ministère des Forêts, de la Faune et des Parcs du Québec Québec, QC, Canada; ^3^Direction de la Recherche Forestière (Forest Research Branch), Ministère des Forêts, de la Faune et des Parcs du Québec Québec, QC, Canada; ^4^Department of Plant Sciences, University of Oxford Oxford, United Kingdom; ^5^Canada Research Chair in Forest Genomics, Centre for Forest Research and Institute for Systems and Integrative Biology, Université Laval Québec, QC, Canada

**Keywords:** genomic fingerprint, tissue culture, quality control, somatic embryogenesis, parental assignment, breeding program, traceability, SNP

## Abstract

Biological material is at the forefront of research programs, as well as application fields such as breeding, aquaculture, and reforestation. While sophisticated techniques are used to produce this material, all too often, there is no strict monitoring during the “production” process to ensure that the specific varieties are the expected ones. Confidence rather than evidence is often applied when the time comes to start a new experiment or to deploy selected varieties in the field. During the last decade, genomics research has led to the development of important resources, which have created opportunities for easily developing tools to assess the conformity of the material along the production chains. In this study, we present a simple methodology that enables the development of a traceability system which, is in fact a by-product of previous genomic projects. The plant production system in white spruce (*Picea glauca*) is used to illustrate our purpose. In Quebec, one of the favored strategies to produce elite varieties is to use somatic embryogenesis (SE). In order to detect human errors both upstream and downstream of the white spruce production process, this project had two main objectives: (i) to develop methods that make it possible to trace the origin of plants produced, and (ii) to generate a unique genetic fingerprint that could be used to differentiate each embryogenic cell line and ensure its genetic monitoring. Such a system had to rely on a minimum number of low-cost DNA markers and be easy to use by non-specialists. An efficient marker selection process was operationalized by testing different classification methods on simulated datasets. These datasets were generated using in-house bioinformatics tools that simulated crosses involved in the breeding program for which genotypes from hundreds of SNP markers were already available. The rate of misidentification was estimated and various sources of mishandling or contamination were identified. The method can easily be applied to other production systems for which genomic resources are already available.

## Introduction

In agriculture and other industry sectors, traceability of the biological material has become mandatory to assess quality control and to trace material upstream and downstream of the value chain. Phytosanitary issues such as the mad cow disease crisis have certainly contributed to stress the importance of tracing living material (Smith et al., 2005). More recently, economic incentives, rather than government regulation have led industries to implement such procedures (Golan et al., 2004). While traceability systems can improve food safety, they also incontestably translate in to enhanced enterprise performance (operating efficiencies) and higher profits (Golan et al., 2004).

Traceability systems that use byproducts of genomics, for instance genetic markers, figure among the various ways that such a quality control system can be applied. Traceability systems appear to be particularly relevant in fields where genomic information is already available, which saves time and money (e.g., olive oil, grapevine, and sheep in Agrimonti et al., 2011; Cabezas et al., 2011; Clarke et al., 2014, respectively). Similarly, quality control systems that rely on genetic markers are increasingly being used for various scientific research purposes in order to authenticate large reference living collections or before starting any new valuable experiment that relies on specific genotypes [e.g., in grapevine (Pollefeys and Bousquet, 2003), *Mycobacterium tuberculosis* (Filliol et al., 2006), and the plant model *Arabidopsis thaliana* (Simon et al., 2012)].

Over the last decade, many non-model species have benefited from the advent of the so-called genomic era (Ekblom and Galindo, 2011; Ellegren, 2014). A vast diversity of agronomic crops, farm animals as well as fish, and forest trees species populations/varieties are now being managed, and improved with the help of genomic tools. Greater affordable access to sequencing technology has led to the development of many research programs for an array of species and, indirectly, to the production of a large amount of genomic resources, including single nucleotide polymorphism (SNP) markers. This has created new challenges, but also new opportunities (EMBL-EBI, 2014).

White spruce (*Picea glauca*), one of the most planted forest tree species in Canada, has become central to genomic research programs in the last decade (*Arborea* and *SmartForest*), and is the current focus of major genomic investments in advanced breeding programs in Canada (*FastTRAC* Project). As for many breeding programs, white spruce improvement relies on the production of offspring produced through controlled crosses among many elite trees. The scheme of production for white spruce elite varieties to be adopted by Quebec nurseries consists of the large-scale production of white spruce seedlings (up to 2 million/year) through somatic embryogenesis (*in vitro* culture/tissue culture) in combination with rooted cuttings. This is seen as the most efficient means to increase genetic gains (up to 35%), but also to decrease rotation age (by up to 10 years); in other words, more volume in less time (Petrinovic et al., 2009; Gélinas and Bull, 2013). The use of a clonal multiplication process makes it possible to canalize 100% of the measured gain and allows the production of the desired trees *ad infinitum*. However, it would also reproduce, as effectively, a non-desirable tree that would have been the result of technical or human error.

In any living production system with large-scale operations, there is a degree of uncertainty associated with human error, which is likely proportional to the number of steps required to obtain the final product (e.g., seedlings from elite varieties). In white spruce, the seedling production strategy is no different from other systems and consists of a multi-stage procedure. First, breeding activities, which are very labor-intensive because they are achieved within a narrow time frame, are subdivided in to different sub-activities: seed orchard establishment with grafted materials, pollen collection, controlled crosses, cone collection, seed extraction, and seed storage. These multiple steps are then followed by somatic embryogenesis (SE), which also consists of four main stages: initiation, growth (including cryopreservation), maturation of the somatic embryos, and germination (Klimaszewska et al., 2016). In the absence of a quality control system throughout this whole plant production process, it is difficult to guarantee that the expected improved productivity of the planted stock will be achieved 40 years after planting. Preferably, a traceability method should be developed and implemented upstream of the production process in order to prevent the introduction of errors from the very beginning.

Extensive genomic resources, including genetic markers and SNP genotyping data, have been developed for white spruce and some of its elite trees (Pavy et al., 2013a,b). This allowed for the investigation into the technical feasibility of a traceability system for white spruce somatic plant production. In the current tree production system, traceability has to take into account two main aspects: (i) the detection of past errors; and (ii) the prevention of future ones. Hence, there is a need to develop a unique and affordable SNP array system that can verify the breed of origin and that can produce a unique fingerprint for each embryogenic cell line.

In this study, we will present the different steps involved in the development of a traceability system using white spruce as a case study. We will demonstrate that the use of existing genomic resources in combination with simulations makes it possible to build a system that is cost-effective, reliable and simple. These qualities should make it possible to build similar systems for other species and programs that have to manage collections of living material and/or that use clonal multiplication techniques.

## Materials and methods

### Production of high-value white spruce trees

The development and testing of the traceability system were conducted using a large part of the white spruce (*P. glauca* [Moench] Voss) advanced breeding population managed by the Ministère des Forêts, de la Faune et des Parcs (MFFP) of the province of Quebec, which consists of a subset of 73 (superior) elite trees that were selected by the MFFP for seedling production using somatic embryogenesis (SE).

**Figure 1:**
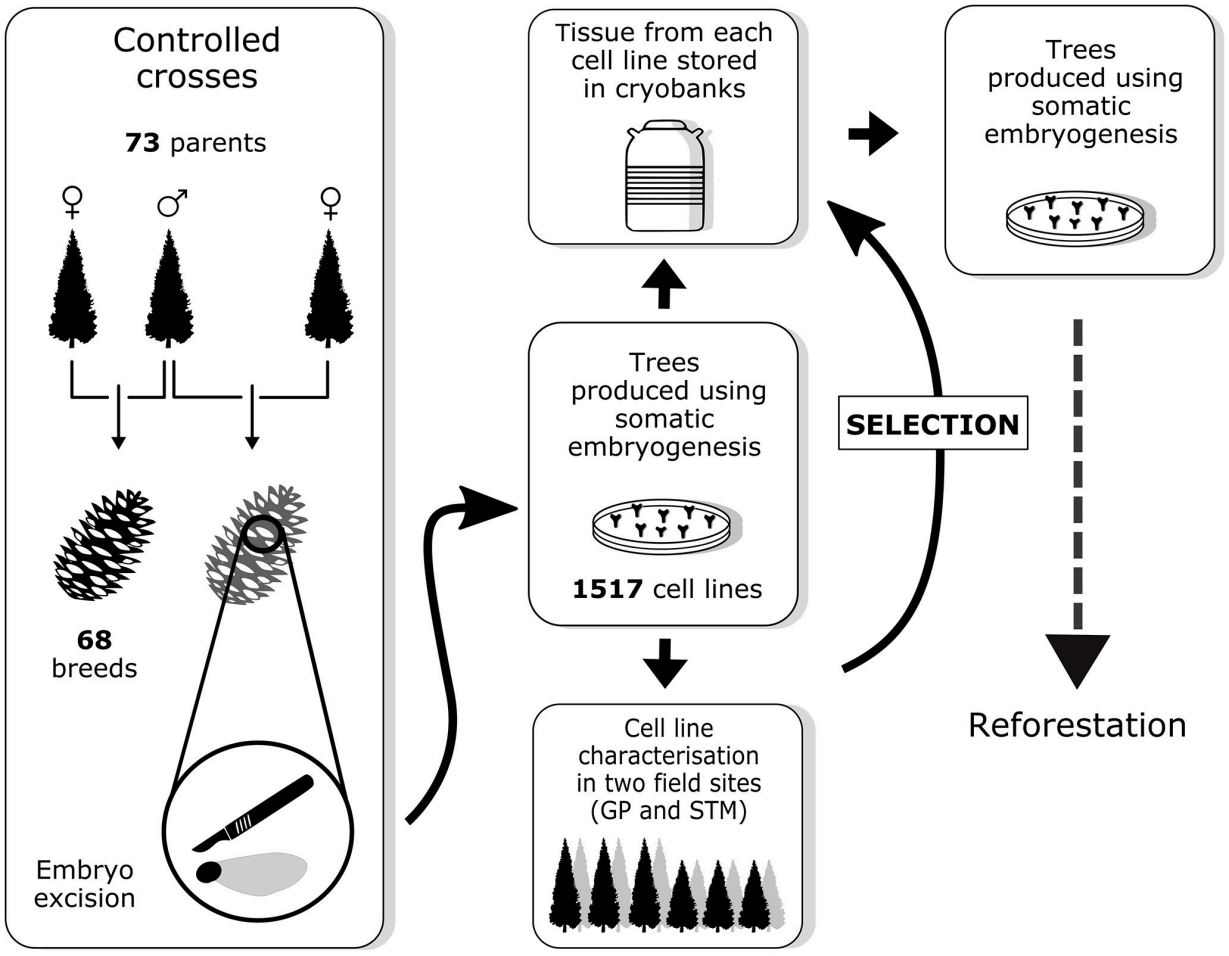
Overview of the breeding strategy for white spruce as described in this paper. Field sites abbreviations: GP, Grandes-Piles; STM, St-Modeste.

Sixty-eight controlled crosses were conducted among the 73 parental elite trees between 2004 and 2013 (Figure 1). From these controlled crosses, 1,517 embryogenic cell lines (hereafter referred to as cell lines) were obtained through SE. For each cell line, embryogenic tissue was cryopreserved and SE-derived plants (somatic plants) were produced at the St-Modeste forest nursery. Between 8 and 16 trees per cell line were planted in two field tests near two MFFP seedling production centers (in Quebec at St-Modeste, 47°50′N, 69°30′W; and Grandes-Piles, 46°41′N, 72°43′W) between 2007 and 2016. After 8–10 years of growth, a subset of cell lines will be selected by the MFFP based on the performance (combining superior growth and other desired characteristics) of the corresponding somatic plants. After this selection, the *modus operandi* will consist of the production of trees from these cell lines by the nursery. This implies that once the individual selection has been made, the cell lines can be removed from cryopreservation where they have been stored for several years during which time the tree characteristics were being observed. Trees from the selected cell lines can be produced through a second round of SE, from the multiplication of embryogenic tissue to maturation of somatic embryos, which in turn can be quickly germinated and multiplied through rooted cutting production (Tremblay and Lamhamedi, 2006).

### Preliminary groundwork: marker selection from available datasets

We already had genotype information on 742 biallelic SNPs drawn from a previous genomic project (Pavy et al., 2013b). From those 742 SNPs, a subset of 458 candidate SNPs for traceability was delineated using two preliminary quality criteria: (1) no missing data for any of the 73 parents; and (2) the associated GC score for each SNP (quality score associated with Illumina Infinium iSelect® genotyping assay) had to be equal or superior to 0.60.

### Building the traceability system

#### Step 1: identifying constraints: number/type of informative markers and genotyping technology

**Figure 2:**
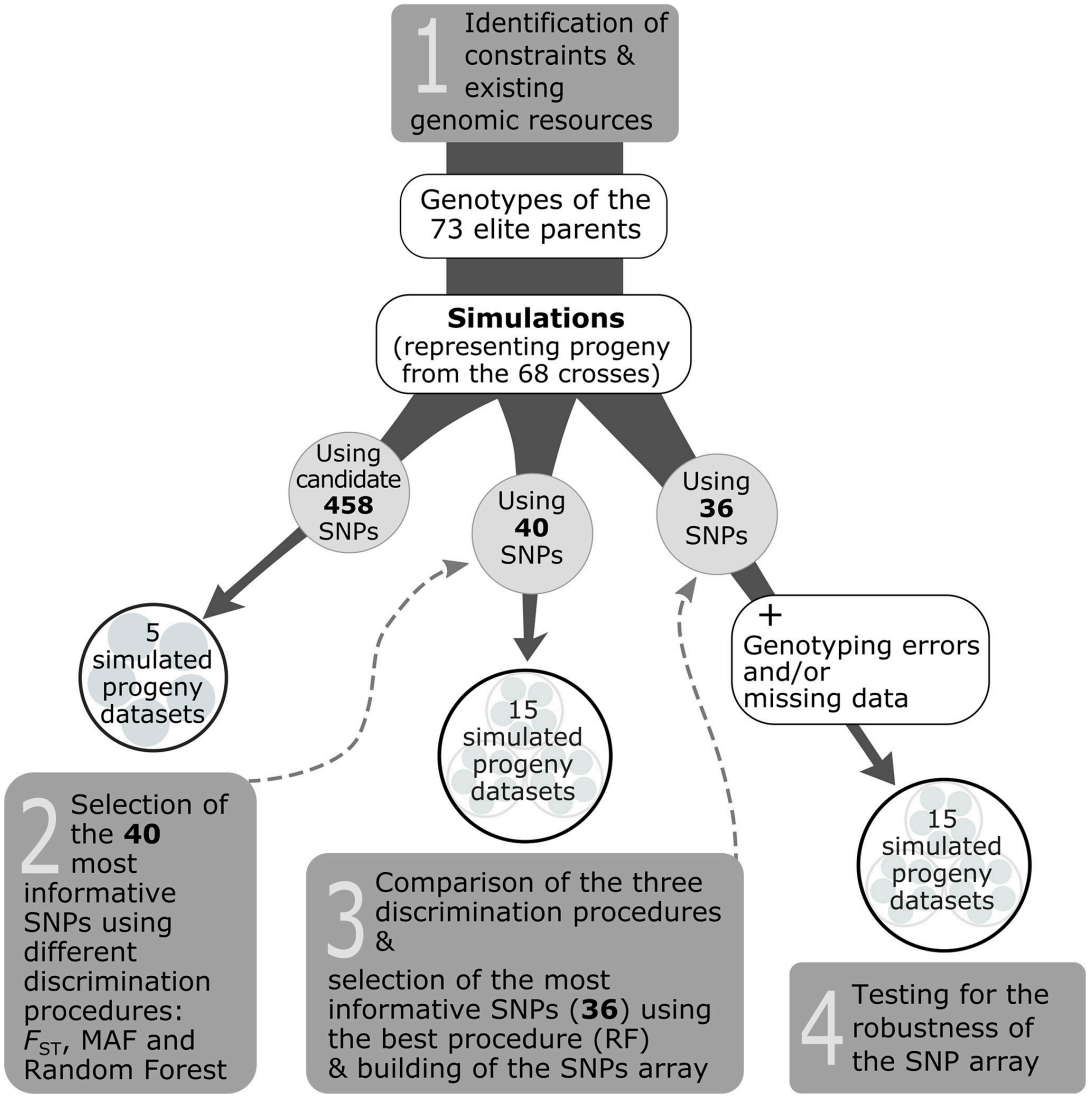
Overview of the strategy used to develop a traceability system for the white spruce case study (steps 1–4).

The selection of the genotyping assay depended on the type of markers available (here, SNPs). While several SNP genotyping technologies and platforms exist, the Sequenom® iPLEX® Gold Technology (hereafter referred to only as Sequenom®) was used because it makes it possible to build SNP arrays with a modest number of markers that are affordable for end-users (nurseries), and also because commercial genotyping platforms using this technology are available and make obtaining genotypic information easy for end-users. In addition, this technology is less restrictive in terms of quality and quantity of template DNA. Based on this, our objective was to select the 40 most informative SNPs among the subset of 458 candidate SNPs, that corresponded to the capacity of no more than a single Sequenom® genotyping array (step 1 in Figure 2).

#### Step 2: generating simulated progeny genotype datasets and application of three different discrimination procedures

To delimitate the set of 40 most informative SNPs for the Sequenom® assay, the general strategy was to perform marker selection from progeny-simulated genotypes (hereafter called simulated progeny; Figure 2). Such a procedure was seen as cost effective and time efficient compared to genotyping the existent progeny with the 458 candidate SNPs for the sole purpose of delimitating the set of most informative SNPs, especially given that these simulated progeny datasets should resemble the genotypes of cell lines (actual progeny) of the white spruce elite breeding program. To obtain simulated progeny, an in-house bioinformatic script was created to generate the progeny genotypes based on genotypic information available from the 73 elite parents, and following the mating scheme involving the 68 controlled crosses conducted between these parents in the elite breeding program. The script was adapted from the commonly used Hybridlab software (Nielsen et al., 2006), in which, for any given cross, an allele from each parent and for each marker is randomly selected to generate the offspring genotype following Mendelian expectations. To develop the whole traceability system, we built a total of 35 simulated datasets that we used to carry out three different steps (see steps 2, 3, and 4 in Figure 2). For each simulated progeny dataset, 100 offspring per cross for each of the 68 controlled crosses were generated.

The first five simulated datasets were produced using the 458 candidate SNPs for which all elite parents were genotyped. Those five datasets were used to test for the best discrimination procedure to select the 40 most informative markers. Three procedures were tested: *F*_st_, MAF, and Random Forest (step 2 in Figure 2). The first consisted in keeping the 40 markers harboring the highest *F*_st_-values estimated among the 68 crosses or full-sib families. For the second procedure, we selected the 40 markers showing a minimum allele frequency (MAF) close to 0.5 (i.e., maximal MAF). The third procedure relied on the Random Forest classification algorithm (Breiman, 1999) which uses a re-sampling procedure to put the SNPs in order according to their importance in explaining the variance among the different crosses. Then, the 40 most informative markers were selected using the Gini index, where a high Gini indicates that a particular predictor (here a SNP) plays a greater role in partitioning the data into the defined classes and thus in discriminating the crosses. The Random Forest analysis was performed in the R environment using the Random Forest package (v.4.6–12; Liaw and Wiener, 2015). Ten thousands classification trees were grown; otherwise, default values of the *randomForest* R function were used.

#### Step 3: identification of the best discrimination procedure for the selection of the most informative SNPs

The capacity of each discrimination procedure described above to correctly assign progeny to their cross of origin was assessed using a second ensemble of simulated progeny datasets (step 3 in Figure 2). Five new datasets were built for each of the 40 SNPs identified to be the most informative by each of the three discrimination procedures in the previous step (*F*_st_, MAF, and Random Forest), for a total of 15 simulated progeny datasets.

For this purpose, two assignment methods were used: (1) FAP, which uses an exclusion-based analysis for parental assignment (Taggart, 2007); and (2) Papa, a parental allocation software that implements a likelihood-based method to identify the cross of origin (Duchesne et al., 2002). In FAP, the “Allele Mismatch Tolerance” was set to one allele in order to allow imperfect matches to be identified in cases where no match was found for the full 458-SNP dataset. For both assignment methods, an input file representing the 73 elite parents and 68 crosses between them was used.

The discrimination procedure presenting the best assignment performance using the simulated progeny datasets and altered simulated progeny datasets was then used to choose the 40 most informative SNPs and build the Sequenom® SNP array. Also, SNPs were selected from as many distinct genes as possible in order to minimize linkage between markers using data from the gene catalog developed for white spruce (Rigault et al., 2011).

#### Step 4: testing the robustness of the selected most informative SNPs

To verify the robustness of the 40 SNP array with the most informative SNPs to correctly assign offspring to their cross of origin, new bioinformatics scripts were created to add a controlled number of missing data and genotyping errors to the simulated progeny datasets. The percentages of missing data or genotyping errors generated by our in-house program were distributed randomly among the simulated progeny and markers of the datasets. Five simulated progeny datasets (built using the 36 working SNPs of the Sequenom® array, see Results Section) were submitted to the program to create 15 altered datasets presenting different percentages of missing data (3.5, 7.0, 10.0, 13.5, and 17.0%), genotyping errors (0.5, 1.0, 2.0, 3.0, 5.0%) and a combination of the two (missing data/genotyping errors: 3.5/0.5, 7.0/1.0, 10.0/2.0, 13.5/3.0, 17.0/5.0%; step 4 in Figure 2).

The two same assignment methods, FAP and Papa, were then used on the 15 altered simulated progeny datasets using the same conditions as those described above, in order to test the robustness of the selected SNP array (step 4 in Figure 2).

### Implementing the traceability system in white spruce

#### Sampling

We minimized the risk of introducing sampling errors in our experiment by paying special attention to sample collection and preparation. A systematic approach was used in which, for instance, each sample was numbered in the field following its predetermined field position so that sampling errors could be more easily traced.

During autumn 2013 and summer 2015, fresh needles from all cell lines planted at the St-Modeste test site were collected. During summer 2014, we completed the sampling in the second test located in Grandes-Piles. We collected fresh needles from cell lines that had not previously been sampled in St-Modeste and for lines that presented incongruities following preliminary analyses of the St-Modeste collection. A copy of each of the 1,509 lines stored as embryogenic tissue in liquid nitrogen was also sampled in two cryobanks (replicates) located at the St-Modeste forest nursery and at the MFFP scientific headquarters in Québec.

#### Sample preparation, DNA extraction and genotyping

Before DNA extraction, the samples from embryogenic cell lines stocked in cryogeny had to be cleaned by removing the cryoprotectant DMSO. Embryogenic tissue samples stored in cryopreservation were thawed in 37°C water for 2 min. DMSO was removed by centrifuging for 6–10 min at 5,250 rpm and discarding the supernatant. Cells were cleaned by re-suspending the pellet in 500–750 μL of PBS 1x buffer, transferred to eight-strip-tubes, and centrifuged at 5,250 rpm for 10 min. The supernatant was removed as described above and the pellet was stored at −20°C until DNA extraction.

DNA was extracted from fresh needles and embryogenic tissue using the Nucleospin 96 Plant II kit (Macherey-Nagel Inc., Bethlehem, PA) following the manufacturer's protocol for vacuum processing with the following modifications: (a) cell lysis using buffers PL2 and PL3 (PL2 was heated for 2 h at 65°C instead of 30 min), and (b) elution with an in-house Tris-Cl 0.01 mM pH 8.0 buffer.

All samples (collected as described above, including the 73 elite parents involved in the controlled crosses) were genotyped using the 40-SNP Sequenom® array developed in this project. Genotyping was performed at the Génome Québec Innovation Centre (McGill University, Montreal, QC) using their internal protocols. In total, after removing individuals presenting more than 15% of missing data, genotypes were obtained for 2,845 trees and 1,501 tissue samples, for a total of 4,346 samples representing 1,517 cell lines (**Figure 4**). Allele frequencies and summary statistics for all SNPs were calculated using Cervus v.3.0.7 (Marshall et al., 1998). To certify the cross of origin of those 4,346 samples, they were analyzed using the same FAP and Papa assignment methods mentioned above, and using the same options as for simulated progeny datasets (see above). Moreover, multilocus fingerprints for each individual (embryogenic tissues and trees) were generated by the concatenation of all single genotypes for each individual from each cell line.

## Results

### Development of a traceability system: key elements to consider

#### Choosing the most efficient discrimination procedure for informative SNPs

The selection of the most informative markers was done using simulated progeny datasets [five datasets containing genotypes for 6,800 individuals (at a rate of 68 crosses and 100 progeny per cross) and for the 458 candidate SNPs; step 2 in Figure 2]. Results obtained from parentage assignment software FAP and Papa were used to select the most efficient discrimination procedure for identifying informative SNPs among the three procedures tested: Random Forest, Minimum Allele Frequency (MAF), and *F*_st_ (step 3 in Figure 2). The Random Forest procedure performed the best as it correctly assigned all genotyped individuals using both the FAP and Papa assignment softwares. The MAF procedure ranked second as the 40 selected markers got a low percentage of errors from assignment softwares (0.06% using FAP and 0.03% using Papa). The procedure based on *F*_st_ was the least efficient (0.47% error rate using FAP and 0.50% using Papa). Interestingly, only six SNPs were shared among the 40 most informative ones by the three procedures tested. Following these comparison results, the set of 40 most informative SNPs used to develop the Sequenom® genotyping array was identified with the Random Forest procedure, and additional most informative SNPs to satisfy the probe design of the Sequenom genotyping assay were also drawn from this procedure (see below).

#### Developing a SNP array for genotyping at the operational level

The transfer of SNPs shown to be successfully genotyped from a previous technology platform (Illumina Infinium iSelect® technology) to a new one (the present Sequenom® technology) that requires longer flanking regions surrounding a SNP had to be taken into account for the final design and selection of SNPs to construct the new genotyping array. Consequently, we had to choose 40 SNPs out of the best 61 (according to the Random Forest procedure) that had adequate flanking sequences, rather than simply select the first 40 best ones. Indeed, the previous technology (Illumina Infinium iSelect® technology) used to generate the white spruce genotypic data from which the 458 candidate SNPs were selected relied on shorter oligonucleotide probes (50 mer) compared with the longer ones of the Sequenom® assay (100–150 mer). For this reason, the latter technology is more sensitive to the occurrence of any polymorphism flanking the SNPs.

The assembled 40 SNP array was then used to genotype a total of 4,346 samples. Overall, 36 out of 40 SNPs were successful, 2 SNPs failed, another one was monomorphic, and the last one showed inconsistent results (Table 1). Of the 4,346 samples genotyped using this SNP array, the percentage of missing data per SNP ranged from 1.1% (AA-019; ss538953749) to 8.3% (AA-015; ss538943850), with an average of only 2.4% estimated over all SNPs. Using replicated control samples, the reproductibility rate of the assay was 99.9% over all successful SNPs. Indices representing the MAF value of each SNP among all samples, polymorphic information content (PIC), heterozygosity, and combined non-exclusion probability (for a candidate parent pair) for each SNP are presented in Table 1. In parentage analysis, the non-exclusion probability corresponds to the average probability of not excluding a single randomly-chosen unrelated individual from parentage (Jamieson and Taylor, 1997; Marshall et al., 1998). When all SNPs were combined, the probability of non-exclusion for our dataset was estimated at 3.1 × 10^−5^, indicating that the designed SNP array was very unlikely to wrongly identify an unrelated tree as one of the elite parents.

**Table 1:** Summary statistics for each SNP locus of the Sequenom® genotyping array.

**Locus**	***SNP name (NCBI)***	***H*_o_**	***H*_e_**	**PIC**	**NE-PP**
A-001	ss538953861	0.404	0.484	0.367	0.724
A-002	ss538943407	0.204	0.280	0.242	0.800
A-003	ss538953915	0.412	0.455	0.352	0.733
A-004	ss538944742	0.399	0.491	0.371	0.722
A-005	ss511222774	0.507	0.495	0.374	0.718
A-006	ss524300150	0.288	0.338	0.281	0.776
A-007	ss538941338	0.007	0.007	0.007	0.993
A-008	ss538942949	0.474	0.484	0.367	0.724
A-009	ss538954305	0.396	0.407	0.324	0.749
A-010	ss538943025	0.494	0.491	0.371	0.722
A-011	ss511222888	0.228	0.255	0.223	0.814
A-012	ss538940594	0.448	0.498	0.374	0.719
A-013	ss538942166	0.447	0.498	0.374	0.719
A-014	ss538954139	0.432	0.447	0.347	0.736
A-015	ss538943850	0.437	0.500	0.375	0.719
A-017	ss538953932	0.376	0.436	0.341	0.740
A-019	ss538953749	0.525	0.465	0.357	0.730
A-020	ss538941729	0.368	0.365	0.299	0.765
A-021	ss538942910	0.510	0.492	0.371	0.721
A-022	ss538944651	0.465	0.439	0.342	0.739
A-023	ss538942912	0.514	0.492	0.371	0.721
A-024	ss538941273	0.456	0.493	0.372	0.721
A-025	ss538946011	0.374	0.403	0.322	0.751
A-026	ss538951794	0.427	0.418	0.331	0.746
A-027	ss538945503	0.433	0.459	0.353	0.732
A-028	ss538940804	0.438	0.483	0.366	0.724
A-029	ss538945192	0.428	0.429	0.337	0.742
A-030	ss538951576	0.507	0.477	0.363	0.726
A-031	ss538940956	0.486	0.474	0.362	0.727
A-033	ss538944911	0.478	0.476	0.363	0.726
A-034	ss538945049	0.428	0.465	0.357	0.730
A-036	ss538942739	0.085	0.081	0.078	0.927
A-039	ss538945302	0.302	0.299	0.254	0.793
A-040	ss538954082	0.385	0.405	0.323	0.750
A-041	ss538940617	0.395	0.407	0.324	0.749
A-042	ss538944866	0.533	0.498	0.374	0.719

*H_o_, observed heterozygosity; H_e_, expected heterozygosity; PIC, polymorphic information content; NE-PP, average non-exclusion probability for a candidate parent pair*.

The regenotyping of the parents for the same set of SNPs, but using a new genotyping technology, allows comparisons to be made between both genotype datasets. For three parent trees [CUS-4(77109); CAR-8(93118); and CAR-10(93121)], large differences were noticed (23/36; 21/36; 17/36, respectively). Manual verification of those trees was done by inferring their genotypes using the genotypes of their progeny; it led to the conclusion that their genotypes obtained from Sequenom® were the correct ones and that the differences observed were likely caused by genotyping the wrong trees as a result of laboratory misidentification or mislabelling in the previous large-scale genotyping project (Pavy et al., 2013b). Moreover, for one locus (AA-033), 13/73 individuals presented a different genotype, which likely indicates the genotyping of a different SNP from a paralogous gene from the same gene family (paralogous SNP). Over the 70 identical parents, 2.3% of the genotypes were different between the Sequenom® and Illumina Infinium iSelect® genotyping technologies.

#### Testing the robustness of the newly built 40 SNP genotyping array

**Figure 3:**
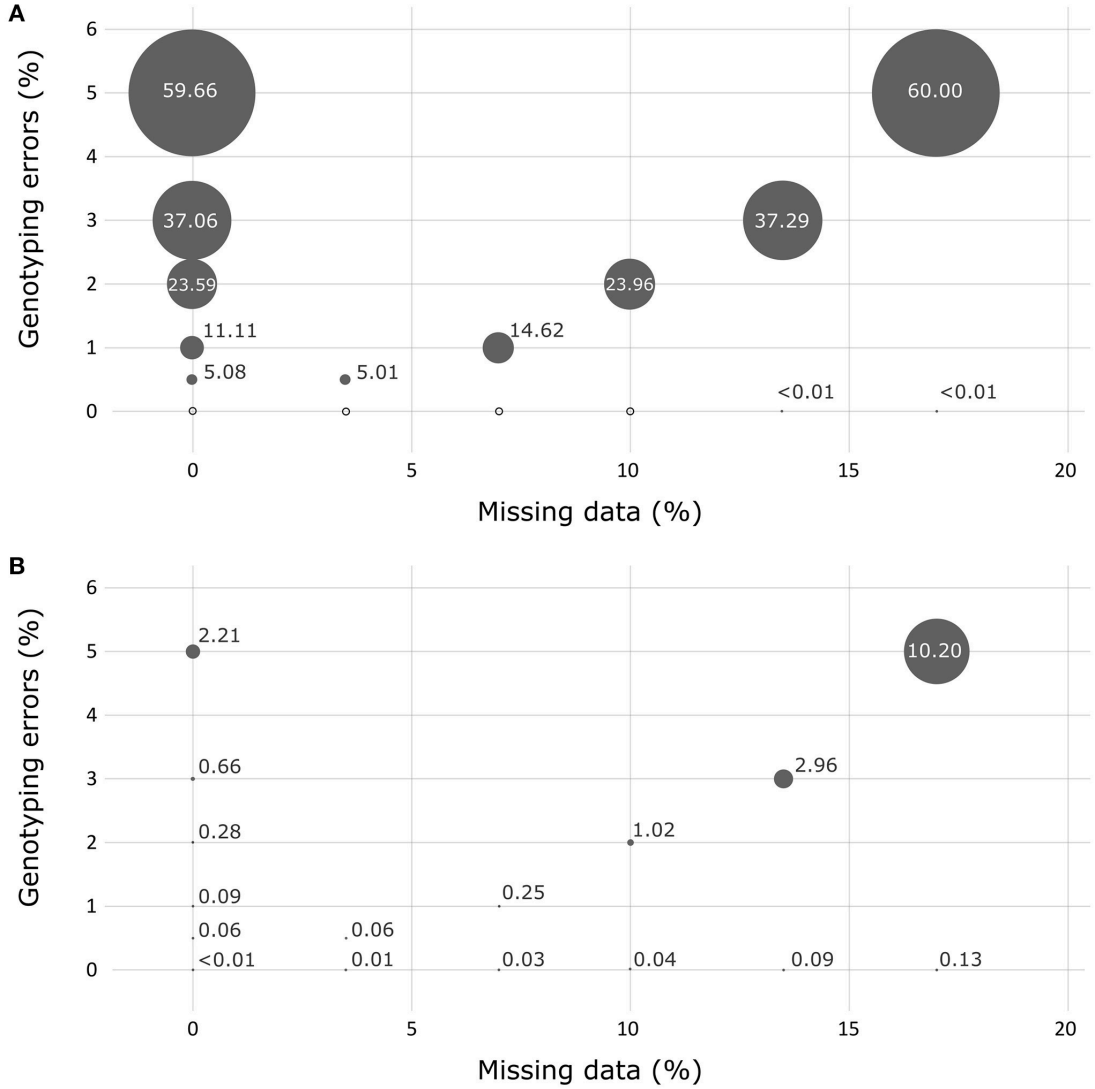
Assessment of the robustness of the assignment exclusion method FAP **(A)** and of the parental allocation method Papa **(B)** for detecting the presence of missing data and/or genotyping errors using simulated progeny datasets. Circle size represents the percentage of errors measured.

In the absence of missing data or genotyping errors, the genotypes obtained for the simulated progeny datasets using the 36 successful SNPs of the Sequenom® array showed no errors when using the FAP assignment method (Figure 3). The results obtained with the Papa assignment method showed a very low percentage of errors for two crosses (0.1 and 0.3% for crosses 2,422 and 2,402, respectively). When testing simulated progeny datasets presenting genotyping errors, Papa was more robust than FAP. For instance, when a percentage of 0.5% genotyping errors was introduced over the whole dataset, Papa resulted in a percentage of wrong assignments that varied between 0 and 0.8% (average of 0.1%), while it varied between 0.3 and 8.7% (with an average of 5.1%) using FAP. Conversely, when the occurrence of missing data was tested alone, the use of the FAP software resulted in the highest robustness, with a maximum level of false assignments not exceeding 0.01%. The use of Papa showed that crosses that had one of the two parents in common were, not surprisingly, more afflicted by wrong assignments (data not shown). Globally, the effect of missing data appeared negligible for FAP. In fact, we observed about the same overall performance for this analysis when we tested the effect of genotyping errors only or the combined effects of genotyping errors and missing data.

### White spruce traceability system: from theory to empirical evidence

#### Detecting past errors: assignment of embryogenic cell lines to their cross of origin

**Figure 4:**
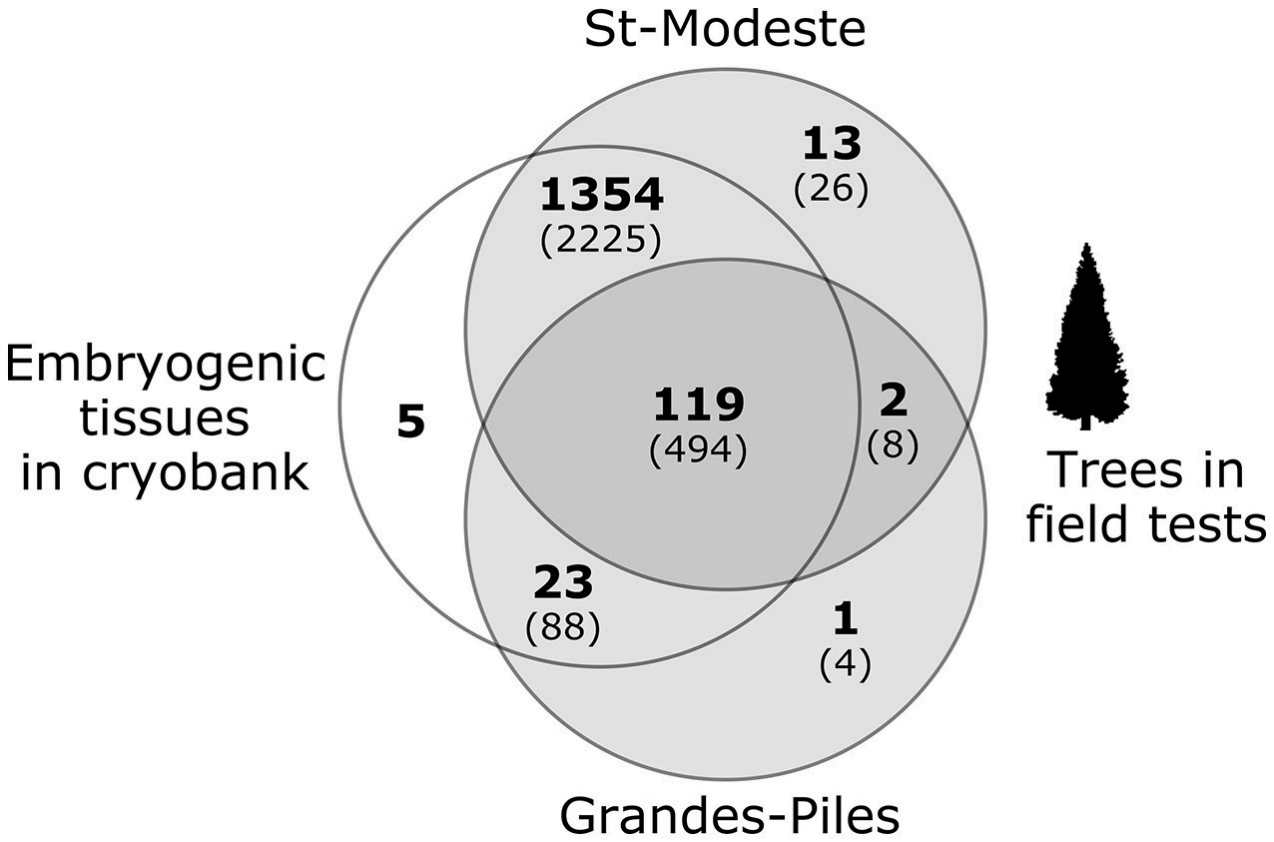
Number of cell lines genotyped and analyzed in this project (in parentheses, the number of trees sampled). Each circle represents one type of material: on the left, embryogenic tissues in cryobanks and on the right, trees in two separate field tests.

Using both the FAP and Papa assignment methods, the 4,346 genotypes (1,517 lines; as reported in Figure 4) were first assigned to one of the 68 crosses (between 73 parents).

The results obtained with FAP made it possible to check whether or not an individual belonged to its putative cross of origin. When a bad assignment was obtained, we used Papa to check if the problem came from the mother, the father, or from both parents (Table 2). The use of both assignment methods allowed us to take advantage of the great power of the exclusion-based FAP method and of the precision of the Papa method to identify the problematic parent, so as to facilitate the identification of potential sources of errors and investigate how these errors might have happened.

**Table 2:** Types and frequency of the different errors found among the individuals and stages analyzed.

**Stage**	**% of 112 problematic cell lines**	**Diagnostic of error**	**Likely cause**
Controlled crosses	43.7	Correct mother/wrong father	Pollen contamination
Controlled crosses	21.4	Wrong mother and sometimes wrong father	Misidentification of parental trees in the seed orchard
Somatic embryogenesis laboratory, cutting production or planting	30.2	Correct embryogenic tissue in cryobanks, but errors found among trees in the field	Misidentification of the material
Cryobanks	4.8	Wrong embryogenic tissue, but correct trees planted in the field	Misidentification of the embryogenic tissue

*The percentage is the proportion of the 112 cell lines identified as problematic*.

Of the 1,517 cell lines analyzed, 1,405 (92.6%) could be unambiguously assigned to their putative cross of origin. For most of them (with the exception of 25 lines), more than one individual (tree) was analyzed. For 84 of the 112 problematic lines detected (Table 2), all tissues examined, including the embryogenic tissues in cryobank and corresponding trees, were identical; this indicates that the errors occurred upstream of the multiplication process, presumably at the level of the controlled crosses. For 22 of those problematic lines, the tissue from the cryobank was not problematic, but at least one of the trees was. This indicates that errors likely occurred after the step of the controlled crosses. For the remaining six lines, the trees in the field were legitimate, but the embryogenic tissue from the cryobank was not, thus indicating that a possible mix up presumably occurred during the stage of cryopreservation or tissue culture. The use of the Papa assignment method made it possible to identify which parental trees were problematic: 55 lines had the wrong father tree, 16 lines had the wrong mother, and 11 lines have both parental trees wrong.

In a subsequent analysis, for every cell line, we compared the genotypes of trees with their corresponding embryogenic tissue stored in cryopreservation in order to verify if they were identical. The individual comparison of those fingerprints within each cell line also made it possible to identify other problematic samples. The use of fingerprinting allowed us to correctly identify some trees with their corresponding cell lines.

#### Detecting future errors: generating unique fingerprints

The combination of all genotypes from the array of 36 successful SNPs produced a unique fingerprint for the majority of non-problematic cell lines (1,422 lines distributed in 68 breeds), with the exception of 11 pairs of cell lines that shared an identical fingerprint.

## Discussion

The purpose of this work was to show how it is possible to design a reliable in-house traceability system using a modest number of informative SNPs, and how it can be used to detect several sources of misidentification in a multi-step plant production system. The criteria followed for ease of use were: (i) the design step (marker selection) should be easy to understand and follow by non-specialists (e.g., in tissue culture laboratory) and; (ii) the traceability procedure should not be complex to use and interpret by end-users (e.g., in our case, nursery workers). Indeed, with only 36 informative SNPs, we showed, using both simulated and real genotype datasets, that the SNPs selected could be used to test the origin of the trees in a large-scale production system.

### A reliable, simple, and versatile traceability system

SNP markers are well-suited for traceability purposes. Although they are less informative than microsatellites (biallelic vs. multi-allelic), which represent the first markers to be used in identity control systems or in forensics (Weir et al., 2006), SNPs are less prone to genotyping errors (Ball et al., 2010) and they can be multiplexed so that the genotyping process can easily be automated. They also performed better than microsatellites in parentage assignment in wild populations (Hauser et al., 2011). Moreover, microsatellites are also more prone to interpretation errors (Pompanon et al., 2005). For white spruce, the main interest of using SNPs is certainly that they were available from previous genomics projects, as it is the case for an increasing number of species. This project is a good example of how new applications can easily be developed from data mining of existing genomic resources, without the need to make a major investment. It should also be noted that although thousands of markers were available for white spruce from previous large-scale genome projects, in practice, only 742 candidate SNPs were used during the first step of marker selection, not thousands. This shows that there is no need to wait for a large quantity of SNPs (or other markers) to become available before developing a traceability system.

Nevertheless, we experienced unexpected problems when shifting from one genotyping technology platform to another. Therefore, the specificities of any genotyping technology should be taken into consideration when information transfer from one genotyping platform to another is foreseen. In our case, this led us to choose from among the 61 most informative SNPs to design a 40-SNP array, rather than simply pick the 40 best ones. Given that the next best SNPs were also highly informative, they could be used successfully for parentage assignment.

The production of simulated progeny datasets through the use of existing parental genotypic information, and their subsequent use in *F*_st_, MAF, and Random Forest discrimination procedures to identify most suitable SNPs, allowed us to choose the best selection procedure and to identify the most informative SNP markers. In previous studies aiming at selecting markers for parental assignment, the procedure based on MAF of candidate markers was often used (Fernández et al., 2013; Trong et al., 2013; Clarke et al., 2014; Heaton et al., 2014; Liu et al., 2016). Here, we show that the Random Forest procedure led to better results, which could be valuable as it maximized the informative value of the chosen SNPs to build the genotyping assay while keeping to a minimum the number of SNPs required for adequate genetic discrimination of the various cell lines at hand. Indeed, the use of Random Forest to select the best markers maximized the genetic differentiation between crosses from a theoretical *F*_st_ = 0.194 (as estimated with simulated progeny datasets for the original 458 candidate markers available) to an observed *F*_st_ = 0.370 (representing the 1,422 real clonal lines distributed in 68 crosses); however this difference between the *F*_st_ indices obtained with simulated and real data could have been influenced by the number of progeny, which was equal between crosses (*n* = 100) for the simulated progeny datasets and heterogeneous (between 8 and 34 per cross) for the real data. The advantage of Random Forest over the MAF method may appear weak, but should not be considered negligible because other factors may interfere as we observed in our experiment, i.e., the genotyping technology switch that diminished the effective number of markers available and the genotyping technology itself for which a 100% success rate cannot reasonably be expected.

The simulated progeny datasets allowed us to test the robustness of the SNP array for different levels of genotyping errors and missing data, and to compare the capacity of assignment methods to correctly identify the crosses of origin. The exclusion method (FAP) was less sensitive to the presence of missing data than the likelihood method (Papa), although, and as previously demonstrated (Anderson and Garza, 2006), the FAP method was much more sensitive to genotyping errors. This could be explained by the great simplicity of FAP, which is based on the principle of exclusion, and checks the compatibility of offspring and parental genotypes following Mendelian inheritance. We thus chose to use FAP in combination with Papa to obtain a confirmation of the assignment result. Moreover, in cases of wrong assignments, Papa further makes it possible to identify which of the two parents is problematic. This information is valuable for tracing the source of errors (Table 2). Globally, we feel confident about the use of this dual assignment approach given that both were highly congruent (>99%) and that genotyping errors appeared to be very low in our experiment.

Because the use of the 36-SNP genotyping array made it possible to obtain a unique fingerprint for almost every cell line, the analysis of the genotyping results by the final users should be straightforward. Indeed, in order to deliver a convenient tool to end-users, we simply implemented the “allelematch” R package (Galpern et al., 2012) in a user-friendly Web interface using the “shiny” package from the R environment (Chang et al., 2016). The “allelematch” package is dedicated to the identification of multilocus genotypes and has the ability to deal with genotyping errors and missing data. An input file containing the genotypes to be tested is uploaded and compared against the local database containing all the white spruce fingerprints identified in the present project. This local database (a simple text file) can easily be modified by adding or modifying fingerprints for specific lines. Ultimately, the SNP array development certainly needed an expertise in the field of genetics/genomics; however the interpretation of genotypes resulting from its use can be conducted by non-specialists. In this specific white spruce project, the end-users (St-Modeste tissue culture lab's team and managers) were informed on a regular basis all along the project and some of them were involved in the interpretation of the results. At the end of the project, an activity of knowledge transfer was conducted in order to explain the final results and the tools developed to people involved not only in seedling production but also in breeding.

### White spruce case study: practical implications and recommendations

Genotyping the real trees with the 40 SNP array allowed us to identify the putative sources of errors along the value production chain (Table 2). In total, 112 (8.04%) of the cell lines shown to be problematic; meaning that all or some of the tissues tested (within a cell line) were either identified to be an illegitimate progeny or a misidentified tree or embryogenic tissue. The results showed that most errors occurred during the breeding stage, which includes pollen collection and storage, grafting of the parents, and conducting the controlled crosses (Figure 1). For instance, pollen contamination and misidentification of parental trees were found to account for 65% of the misassignments (Table 2). This means that fewer errors occurred during the following stages; which are vegetative propagation in the tissue culture laboratory and establishment of the material in the field. These results indicate that the traceability system can be used only in these two last specific stages, i.e., that a minimal number of control points along the production chain are required. However, it also indicates that particular attention should be paid during the breeding activities, which appeared to be the most critical stage. For example, for the material established in the field in the first year of the program, our results showed that more than one-third of the cell lines were in fact illegitimate progeny with various degrees of contamination (19/47). This highlights the importance of systematically verifying the material obtained from breeding activities upstream of the vegetative propagation and establishment of the material in the field steps. Such recommendations for forest tree breeding programs are not new: almost 30 years ago, Adams et al. (1988) found that around 30% of progeny from controlled crosses in *Pseudotsuga menziesii* and *Pinus taeda* was invalid, and they suggested a verification of the progeny genotypes for all tree improvement programs. Verification of the grafts (vegetative copies of mature trees) in seed orchards has also proven them to often be mislabelled (Wheeler and Jech, 1992). In a recent survey conducted as part of the *Juglans nigra* breeding program, an error rate of 20% was estimated within crosses (among progeny within family), and the family ranking for growth and wood quality traits was quite affected (Zhao et al., 2012). Several studies conducted to verify pedigree integrity in half- or full-sib families have identified pollen as the main source of contamination, at a rate that varied from 4 to 20% (Corley, 2005; Kumar et al., 2007; Doerksen and Herbinger, 2008; Moriguchi et al., 2009; Padi et al., 2015). Although the detection of errors due to pollen contamination may not always bear severe consequences on backward selection in tree improvement programs (Vidal et al., 2015), it is certainly a source of concern in forward selection schemes (Doerksen and Herbinger, 2010; Vidal et al., 2015) Moreover, it has been shown that error misidentification could greatly affect the selection of varieties retained for their resistance to disease or pathogens (Cervantes-Martinez et al., 2006).

Probably the most important challenge in the implementation of a traceability system such as the one described here is to develop a method for which the costs of application will always be lower than the benefits that the system is supposed to deliver or guarantee. The evaluation of such paybacks associated to the production of improved trees, especially long-lived boreal conifers, is difficult to determine with precision mainly because it is hard to evaluate precisely the net benefits of planting improved (and thus more expensive) trees compared with unimproved stock (Petrinovic et al., 2009). Nevertheless, it is safe to assume that the shortening of the rotation age and the genetic gain that is achieved by the use of SE in conjunction with advanced breeding and the possible use of genomic selection schemes (see Park et al., 2016 for a review) may be more or less severely hampered by human error. Studies indicated that using SE in the context of multiclonal forestry can result in genetic gains of as much as 45% for some traits in white spruce (Park, 2002). A simulation study showed that pedigree errors corresponding to a pollen contamination of 10% could decrease the genetic gain expected by about 4% (Israel and Weller, 2000). However, this reduction could be more drastic considering the specific second-generation selection strategy currently planned and being implemented for white spruce in Quebec. Although more than 1,500 cell lines are currently being tested in the field, only a small number (about 50) will be selected and deployed. Such a strategy captures common alleles and maintains genetic diversity while maximizing the genetic gain (Weng et al., 2010; Wahid et al., 2012a). However, the deployment of such a small proportion of the candidate lines may also increase the potential effect of mishandling and misidentifying the material.

Not only could these errors affect the future profitability of the planted forests, but they could also affect other aspects related to research and development given that large clonal tests are also being used in several research projects. As an example, we used the growth measures from 133 clones planted in 2008 in the two field tests, and for which 15% of the lines were problematic, to estimate the effect of those errors on the estimation of clonal heritability (*H*^2^_C_) and family heritability (*H*^2^_F_) (see details of the calculations and results in Supplementary Table S1). Results showed that the presence of errors increased the estimation of *H*^2^_F_ to between 0.073 and 0.172 but had had no effect on *H*^2^_C_ and the estimation of genetic gain. This result is not surprising given that the errors within this subset resulted mostly from controlled cross errors, i.e., from trees that were illegitimate members of their family and that all copies for one cell line were systematically removed when one error was identified within a cell line. Globally, the effect of errors for white spruce was low, but this may not always be the case, as was observed in a cacao tree breeding program where unexpected parentage affected the consistency of the variety's performance for the various traits evaluated (Padi et al., 2015).

Moreover, by comparing the genotypes of the embryogenic tissues maintained in cryobanks to those of trees planted in the field, we also identified some mismatches within 42 cell lines. In a few cases, the genotypes of the embryogenic tissues did not correspond to any of the somatic plant genotypes (Table 2); most of the time, errors originated from the field stage. Such mismatches can certainly compromise the expected gain from multiclonal selection and SE because it supposes that after retrieving the most performant genotypes from the cryogenic banks, the exact same genotypes should be reproduced. The use of fingerprints thus makes it possible to verify this crucial match before going further with the tissue culture multiplication process. Also, our intent was not to develop tools for detecting somaclonal variation, given that it represents a different topic; nevertheless, we recommend that any new phenotype described and declared as a somaclonal variant should be accompanied by its original genetic fingerprint attesting that the cause is not simply due to a mix up of genotypes (Isabel et al., 1993, 1996; Tremblay et al., 1999).

The unique fingerprints generated herein with the aim to evaluate the production process of white spruce somatic plants could also be used to follow planted trees over their rotation age, when planted trees are intermixed with on-site trees from natural regeneration of the same species. Considering that 36 biallelic SNPs can produce 3^36^ (>1.50 × 10^17^) distinct multilocus haplotypes, we can suppose that we can retrieve the uniqueness of the improved trees in the years following planting. This feature can be useful in monitoring which lines better survive and reproduce in the wild. Moreover, such an in-field traceability system can also be used to measure genetic diversity in planted sites, which is an interesting feature given that forest biodiversity is a major concern for population geneticists, foresters, and forest certification organizations especially in the context of climate change (Pawson et al., 2013).

In discussing our results with the end-users and considering that the cost of applying a traceability method using SNPs can be high, we have concluded that the best strategy would be to test only specific samples with our traceability system once the selection step is completed. As an example, we suggest to verify the embryogenic tissues of the selected cell lines when removing the lines from cryopreservation before starting large-scale seedling production. Since it is difficult to estimate the net benefits of using somatic embryogenesis in forest plant production and that our results showed that the errors are rare, we think that this would be the best strategy here. We are also aware that the costs associated with the development and application of such a traceability method constitutes one of the most important criteria for the decision to use it. That being said, although it may be difficult to precisely account for its return on investment, as is the case here, a traceability system would allow one to assess the effectiveness of the system and to highlight the weaknesses in the production chain which is quite relevant in a production system such as the white spruce system. Indeed, in the long term when this material will be deployed there might be unexpected and intangible benefits (forest certification aspects and social licensing) coming from the use of a traceability system in white spruce.

## Conclusions

In this project, three central objectives were achieved: (i) we established an analytical pipeline to design an efficient genotyping tool enabling the traceability of biological materials; (ii) we verified the presence of errors upstream of the multiplication process, i.e., pedigree problems related to breeding activities; and (iii) we generated unique fingerprints for every cell line for traceability purposes during the tree production and deployment process. We showed that a small number of informative SNPs could be used to discriminate efficiently among a large number of full-sib families and track down the illegitimate progeny, and could be used to follow up cell lines and their individuals at different steps of the production and deployment process. Hence, this work highlights the fact that once genomic data associated with the foundation germplasm involved is available, it is quite simple to create simulated datasets representing the variability associated with the putative offspring, and then to identify the most informative markers using a discrimination procedure such as the Random Forest algorithm.

As in the white spruce example presented here, many living production systems use germplasm under the form of elite parents or stock to generate progeny that will form the basis of the exploitation system (e.g., in the dairy cattle, sheep, fish, horticulture, forestry, and agronomic crops industries, or for the use of plant cell lines for vaccine production and other biopharmaceutical compounds). Therefore, a pipeline as shown herein for identifying informative SNPs and then using them for parentage assignment and individual fingerprinting could be useful in those systems to enable traceability at various scales, from research to production.

Not only should traceability be used in closed production systems such as the one presented herein, but tracing the origin could also be applied to fulfill similar demands in alternative markets. Today, consumers value new aspects in their buying choices, such as the country of origin and/or the sustainable aspect related to product fabrication (United Nations Global Compact and BSR., 2014). Hence, such a traceability system could be dedicated to tracking illegal biological material across the world or to certify the origin of a biological product. If those concerns were traditionally oriented toward food products, more and more, concerns about other merchandise should now be addressed. Hence, the method presented here could be adapted with new objectives in mind.

## Author contributions

JG, LT, JM, JB, and NI contributed to the project design; JG, CL, and NI took part in the experimental work; JG and PL performed the statistical and bioinformatic analyses; JG, LT, CL, AR, JB, and NI were involved in both data acquisition and interpretation; JG wrote the manuscript. All the authors contribute in drafting and reviewing the article. All the authors approved the final version of this text.

### Conflict of interest statement

The authors declare that the research was conducted in the absence of any commercial or financial relationships that could be construed as a potential conflict of interest. The reviewer OKH and handling Editor declared their shared affiliation, and the handling Editor states that the process met the standards of a fair and objective review.
