# Supplementary material for: Development of a Traceability System Based on a SNP Array for Large-Scale Production of High-Value White Spruce (Picea glauca)
Source: Front Plant Sci. 2017 Jul 25;8:1264. doi: 10.3389/fpls.2017.01264 (PMC5524734; doi:10.3389/fpls.2017.01264)
Supplement: Supplementary file 1 [file Table1.DOCX]

Supplementary Table S1. Estimates of variance components (σ^2^), heritability (*H*^2^) and genetic gain (ΔG_c_) of growth for white spruce clones measured for total height in the years 2007 and 2009. The estimates were calculated on datasets with and without the errors identified in the present study. When an error was identified within one somatic cell line, all measures taken for this line were removed. In both tests (St-Modeste and Grandes-Piles), a maximum of 16 copies of each line were assessed (planted in 8 blocks). Calculations were done using the methods described in ([Wahid et al., 2012b](#_ENREF_54)) using the REML method in MIXED procedure in SAS v.3.5.

|  |  | **St-Modeste field test** | | | | **Grandes-Piles field test** | | | |
| --- | --- | --- | --- | --- | --- | --- | --- | --- | --- |
|  |  | **Without errors** | | **With errors** | | **Without errors** | | **With errors** | |
| Year of measurement | | **2007** | **2009** | **2007** | **2009** | **2007** | **2009** | **2007** | **2009** |
| Number of families analyzed | | 20 | 20 | 20 | 20 | 19 | 19 | 19 | 19 |
| Number of cell lines analyzed (number of trees) | | 112 (1759) | 112(1759) | 132(2054) | 132(2054) | 111(1725) | 111(1725) | 131(2015) | 131(2015) |
| Family effect | **σ^2^_F_** | 4.346 | 7.618 | 3.343 | 5.802 | 6.350 | 16.138 | 3.965 | 10.434 |
| Clone effect | **σ^2^_C(F)_** | 10.977 | 18.586 | 11.198 | 20.506 | 10.714 | 17.551 | 13.212 | 23.001 |
| Error | **σ^2^_ε_** | 20.644 | 46.260 | 21.386 | 49.423 | 17.963 | 49.122 | 18.690 | 50.226 |
| Phenotypic variance | **σ^2^_Ph_** | 31.621 | 64.846 | 32.584 | 69.929 | 28.678 | 66.673 | 31.902 | 73.227 |
| Clonal heritability | ***H*^2^_c_** | 0.347 | 0.287 | 0.344 | 0.293 | 0.374 | 0.263 | 0.414 | 0.314 |
| Family heritability | ***H*^2^_F_** | 0.348 | 0.283 | 0.270 | 0.210 | 0.522 | 0.495 | 0.350 | 0.344 |
| Genetic gain (%) | **ΔG_c_** | 1.952 | 2.308 | 1.962 | 2.452 | 2.001 | 2.149 | 2.339 | 2.688 |
